# Estimating bystander CPR using a strict documentation-based definition: a multiregional EMS registry analysis

**DOI:** 10.1186/s12873-026-01635-3

**Published:** 2026-06-05

**Authors:** Tariq H. Alshahrani, Ahmed Al-Wathinani, Abdullah Mohammed Alobaid, Matteo Paganini, Mohammed A. Abahussain, Krzysztof Goniewicz

**Affiliations:** 1 Department of Emergency Medical Services, Prince Sultan Bin Abdulaziz College for Emergency Medical Services, King Saud University, Riyadh, 11451 Saudi Arabia; 2 Accidents and Trauma Department, Prince Sultan Bin Abdullaziz College for Emergency Medical Services, King Saud University, Riyadh, 11451 Saudi Arabia; 3 Department of Biomedical Sciences, University of Padova, Padova, 35131 Italy; 4 Polish Air Force University, Deblin, 08-521 Poland

**Keywords:** Out-of-hospital cardiac arrest, Bystander CPR, Emergency medical services, Saudi Arabia, Saudi red crescent authority, Regional disparities

## Abstract

**Introduction:**

Bystander cardiopulmonary resuscitation (CPR) is a key determinant of survival after out-of-hospital cardiac arrest (OHCA), yet engagement remains low and varies across regions. Reliable estimation of bystander CPR prevalence depends on consistent documentation within emergency medical services (EMS) registries. In Saudi Arabia, national evidence on documentation-based prevalence and system-level determinants remains limited.

**Aim:**

To estimate the prevalence of strictly documented bystander CPR, assess data completeness and potential outcome misclassification, examine regional variation, and identify independent predictors of bystander CPR among OHCA cases attended by the Saudi Red Crescent Authority (SRCA).

**Methods:**

We conducted a retrospective cross-sectional analysis of the SRCA OHCA registry (January–June 2024). Bystander CPR was defined conservatively, requiring explicit documentation of CPR initiation prior to SRCA arrival by a non-SRCA individual. Cases lacking clear attribution were classified as unclassifiable. Regional variation was described, and multivariable logistic regression estimated adjusted associations with patient characteristics, witnessed status, EMS response time, and region.

**Results:**

Among 6,378 OHCA records, bystander CPR status was classifiable in 5,271 cases (82.6%), while 1,107 cases (17.4%) were unclassifiable under the strict definition. Among classifiable cases, bystander CPR was documented in 646 cases (12.3%). In the complete-case model (*N* = 4,267), witnessed arrest was strongly associated with higher odds of bystander CPR (aOR = 5.15, 95% CI 4.20–6.31). Longer EMS response time was associated with lower odds (aOR = 0.98 per minute, 95% CI 0.97–1.00), and male sex was associated with higher odds (aOR = 1.26, 95% CI 1.03–1.53). Significant regional disparities persisted after adjustment.

**Conclusion:**

Using a conservative documentation-based definition, bystander CPR engagement in SRCA-attended OHCA cases remains low and uneven across regions. Substantial variation in documentation completeness highlights the importance of standardized reporting for accurate surveillance and benchmarking. Strengthening dispatcher-assisted CPR, expanding population-level training, and enhancing registry harmonization may improve early resuscitation response across SRCA-attended EMS settings in Saudi Arabia.

## Introduction

Out-of-hospital cardiac arrest (OHCA) continues to be a major cause of death worldwide and requires an immediate, well-coordinated response to improve survival [1–2]. The chain of survival depends on rapid recognition of cardiac arrest, timely activation of emergency medical services (EMS), and early initiation of bystander cardiopulmonary resuscitation (CPR), all of which are strongly emphasized in current resuscitation guidelines [1, 3–5]. When initiated promptly, bystander CPR helps maintain blood flow to vital organs, bridging the time until defibrillation and advanced life support can be provided [6–7].

Although early bystander CPR is well established as a life-saving intervention, it remains underutilized in many communities, with participation varying widely across regions. These differences are influenced not only by individual factors such as CPR training, legal and cultural concerns, and confidence in providing assistance, but also by system-level characteristics including local EMS organization and early response processes [8]. Evidence from national and regional improvement initiatives demonstrates that increasing bystander involvement can substantially improve survival outcomes at the population level [9]. Notable geographic differences in OHCA incidence and outcomes further underscore the need for coordinated, system-level strategies to strengthen early response and reduce disparities [10].

Dispatcher-assisted CPR (DA-CPR) represents a practical and scalable strategy to increase bystander CPR initiation, particularly when a witness is present but uncertain how to respond [11]. Broader community-based approaches, such as mobilizing trained lay responders through mobile applications, may also enhance early intervention prior to EMS arrival [12]. However, the evaluation of bystander CPR in real-world settings remains highly dependent on the quality and consistency of prehospital documentation, highlighting the importance of standardized registry-based systems for accurate assessment and quality improvement [13].

In Saudi Arabia, the Saudi Red Crescent Authority (SRCA) serves as the primary EMS provider and has progressively strengthened its capacity to document and evaluate OHCA care across regions. Nevertheless, multicenter EMS registry evidence on the determinants of bystander CPR in Saudi Arabia remains limited and fragmented, particularly with respect to regional variation. A clearer understanding of the factors associated with bystander CPR, and how they differ geographically, is essential for informing targeted interventions aimed at improving early resuscitation response and reducing inequities.

A recent national analysis of the SRCA registry covering the same study period examined the association between bystander CPR and return of spontaneous circulation, with a primary focus on survival outcomes and regional disparities [14]. In that study, bystander CPR was treated as an explanatory variable in survival modelling [14]. However, important methodological questions remain regarding the validity and completeness of bystander CPR documentation within routine EMS records. Specifically, the classification of bystander CPR in registry-based research may be vulnerable to misclassification due to inconsistent pre-arrival documentation, which can substantially influence reported prevalence estimates and regional comparisons. To date, no multicenter EMS registry study in Saudi Arabia has systematically evaluated how a strict, documentation-based definition of bystander CPR influences prevalence estimates, regional variation, and identification of independent predictors within the same EMS registry framework.

This study therefore aimed to (1) estimate the prevalence of bystander CPR using a conservative, documentation-based definition within the SRCA OHCA registry, (2) assess the extent of outcome misclassification and data completeness, and (3) examine independent predictors of strictly documented bystander CPR, with particular attention to regional system-level variation.

## Materials and methods

### Study design

A retrospective cross-sectional analysis was conducted using OHCA registry records from the SRCA covering the period from January to June 2024. This period was selected because it represented the most recent continuously available and fully harmonized registry dataset at the time of analysis.

### Setting and data source

Data were obtained from the SRCA OHCA registry and extracted from monthly datasets spanning January to June 2024. These datasets were merged into a single multicenter EMS registry cohort representing OHCA cases attended by the SRCA across Saudi Arabia during the study period. The registry reflects cases documented within the SRCA EMS system and should not be interpreted as capturing all OHCA events occurring nationally.

### Study population

The study population consisted of OHCA cases attended and transported by the SRCA across Saudi Arabia’s 13 administrative regions. Cases in which CPR was not initiated, such as patients declared dead on EMS arrival, were excluded. The registry did not contain sufficiently detailed operational information regarding the specific criteria used to withhold resuscitation or declare death on EMS arrival. These decisions were made according to routine SRCA clinical practice and may have included obvious signs of irreversible death or other non-resuscitation circumstances documented in the EMS record. Accordingly, the analytical cohort reflects resuscitation-attempted and transport-associated EMS encounters rather than the full national OHCA burden. All remaining eligible cases were included in the analysis.

### Participants and inclusion criteria

All OHCA records captured during the study period were included in the descriptive analyses. For the multivariable regression analyses, only cases with complete information on bystander CPR status and all prespecified predictor variables were included, consistent with a complete-case analytical approach. The reduction from 5,271 classifiable cases to 4,267 complete cases in the multivariable regression analysis was attributable to missing data across one or more prespecified predictor variables included in the complete-case model.

### Outcome measure: bystander CPR

The primary outcome was bystander CPR, defined as a binary variable (Yes/No). Cases were classified as “No” only when registry documentation explicitly indicated the absence of bystander CPR or when no pre-arrival CPR activity was documented despite otherwise complete event documentation, including available information on event chronology and pre-arrival care processes. To minimize outcome misclassification inherent to routine registry data, a strict documentation-based definition was applied. This definition required explicit documentation indicating that CPR was initiated prior to SRCA arrival by a non-SRCA individual. Generic pre-arrival entries lacking clear attribution of the CPR initiator, as well as cases with ambiguous or incomplete pre-arrival documentation, were treated as missing and excluded from outcome classification.

### Predictors

Predictor variables were selected a priori based on established associations with bystander CPR in the OHCA literature and data availability within the SRCA registry. These included patient age, sex, and nationality; whether the cardiac arrest was witnessed; EMS response time; and the administrative region in which the event occurred.

EMS response time was measured in minutes using registry-recorded timestamps and reflects the pre-arrival interval captured within the SRCA EMS response process. Because the study relied on routinely collected registry data, the registry did not provide sufficiently detailed metadata to determine whether this interval uniformly reflected call receipt-to-arrival, dispatch-to-arrival, or another operational time interval across all regions. Nevertheless, the variable consistently represented a system-level pre-arrival response interval recorded within the SRCA registry and was analyzed as a continuous operational measure.

### Data cleaning and harmonization

Monthly datasets were merged, and variable labels and coding were harmonized across files. Regional identifiers were standardized to address spelling and case variations. For regression stability within the strict-classification cohort, Al Bahah, Al Jouf, Najran, and North Border regions were combined into the “Other” category due to sparse counts. Regional analyses were restricted to cases with available and valid harmonized regional identifiers.

### Statistical analysis

Categorical variables were summarized as frequencies and percentages, while continuous variables were summarized as means with standard deviations (SD). Regional proportions of bystander CPR were calculated among cases with classifiable outcomes and presented with corresponding 95% confidence intervals. A multivariable logistic regression model was used to estimate adjusted associations between prespecified predictors and bystander CPR, with results reported as adjusted odds ratios (aORs) and 95% confidence intervals. Model discrimination was assessed using the area under the receiver operating characteristic curve (AUC), in accordance with standard methodological guidance [15]. Statistical significance was defined as a two-sided p-value < 0.05.

### Data completeness diagnostic

To evaluate potential bias related to outcome misclassification under the strict documentation-based definition, we conducted a diagnostic comparison between cases with classifiable bystander CPR status and those categorized as unclassifiable. Differences between groups were examined across key variables, including geographic region, EMS response time, and witnessed status. Categorical variables were compared using chi-square tests, and continuous variables were compared using Welch’s t-tests. This analysis aimed to assess whether missing outcome classification was systematically associated with event characteristics that could influence estimates of bystander CPR prevalence or regional variation.

### Ethical considerations

The study protocol was reviewed and approved by the SRCA ethics committee on October 10, 2023 (NCBE-KACST.KSA: H-01-R-110).

## Results

### Sample characteristics and outcome prevalence

The final merged dataset comprised 6,378 OHCA records. Under the strict documentation-based definition, bystander CPR status could be classified in 5,271 cases (82.6%). Among these classifiable cases, bystander CPR was documented in 646 cases (12.3%), while 4,625 cases (87.7%) did not receive bystander CPR prior to SRCA arrival (Table 1).

**Table 1:** Sample characteristics and outcome classification

Characteristic	Total Dataset (*N* = 6,378)	Strict-Outcome Classifiable (*n* = 5,271)
Bystander CPR = Yes	646 (10.1%)	646 (12.3%)
Bystander CPR = No	4,625 (72.5%)	4,625 (87.7%)
Bystander CPR = Missing/Unclassifiable	1,107 (17.4%)	—

Note. Bystander CPR classification is based on a strict definition requiring explicit documentation of CPR initiation by a non-SRCA actor prior to SRCA arrival

### Data completeness diagnostic

To evaluate potential bias related to outcome misclassification, cases with classifiable bystander CPR status were compared with those categorized as unclassifiable under the strict documentation-based definition (Table 2).

The proportion of unclassifiable cases varied significantly across regions (χ² test, *p* < .001), indicating potential geographic differences in documentation practices. Mean EMS response time was slightly shorter among unclassifiable cases compared with classifiable cases (11.7 vs. 13.3 min; Welch t-test, *p* < .001). No substantial difference in witnessed status was observed between groups.

**Table 2:** Comparison of classifiable and unclassifiable bystander CPR cases under the strict documentation-based definition

Characteristic	Classifiable (*n* = 5,271)	Unclassifiable (*n* = 1,107)	*p*-value
**EMS response time, mean (SD), minutes**	13.3 (8.4)	11.7 (7.6)	< 0.001
**Witnessed arrest, n (%)**	384 (7.3%)	92 (8.3%)	0.214
**Region distribution, n (%)**			< 0.001
Riyadh	535 (10.2%)	124 (11.2%)	
Makkah	1,618 (30.7%)	321 (29.0%)	
Eastern Region	592 (11.2%)	131 (11.8%)	
Madinah	310 (5.9%)	69 (6.2%)	
Asir	231 (4.4%)	54 (4.9%)	
Al Qassim	241 (4.6%)	53 (4.8%)	
Tabuk	125 (2.4%)	27 (2.4%)	
Hail	112 (2.1%)	25 (2.3%)	
Jazan	221 (4.2%)	48 (4.3%)	
Other*	187 (3.5%)	43 (3.9%)	

Note. Differences between groups were evaluated using Welch’s t-test for continuous variables and chi-square tests for categorical variables. Unclassifiable cases refer to records lacking explicit documentation identifying the individual who initiated cardiopulmonary resuscitation prior to SRCA arrival. *“Other” includes Al Bahah, Al Jouf, Najran, and North Border regions. Regional analyses are based on 4,172 classifiable cases and 895 unclassifiable cases with non-missing harmonized regional information

### Regional variation in bystander CPR

**Fig. 1:**
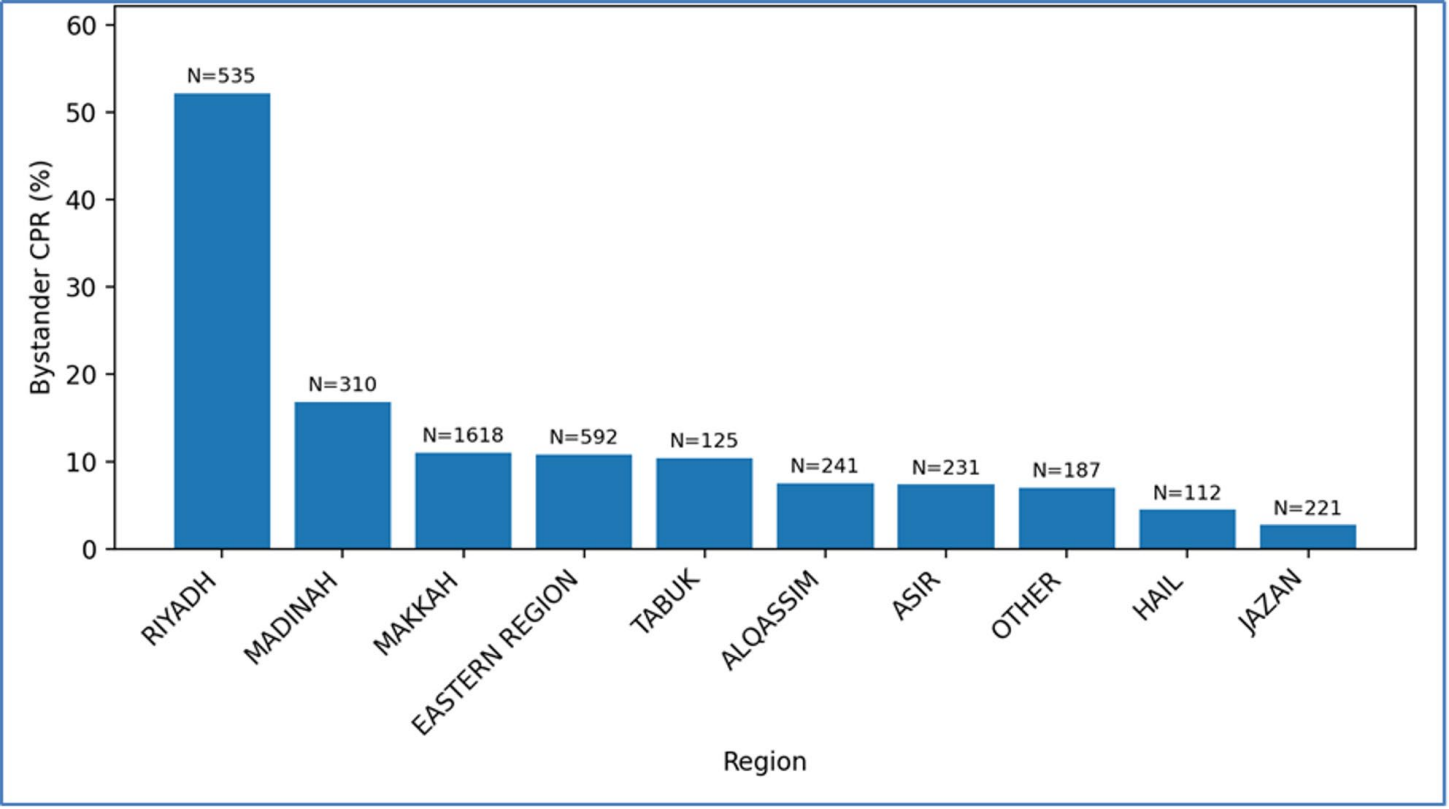
Regional proportion of bystander CPR in OHCA cases (strict documentation-based definition)

Substantial geographic variation in documented bystander CPR prevalence was observed across SRCA-attended OHCA cases in Saudi Arabia (Table 3; Fig. 1). Riyadh demonstrated the highest proportion of documented bystander CPR (52.1%, 95% CI [47.9%, 56.4%]). In contrast, lower rates were observed in several regions, including Jazan (2.7%, 95% CI [1.3%, 5.8%]) and Hail (4.5%, 95% CI [1.9%, 10.0%]).

**Table 3:** Regional distribution of bystander CPR in OHCA cases (strict documentation-based definition)

Region	*N* (classifiable)	Bystander CPR (*n*)	%	95% CI
Riyadh	535	279	52.1	[47.9%, 56.4%]
Madinah	310	52	16.8	[13.0%, 21.3%]
Makkah	1,618	178	11.0	[9.6%, 12.6%]
Eastern Region	592	64	10.8	[8.6%, 13.6%]
Tabuk	125	13	10.4	[6.2%, 17.0%]
Al Qassim	241	18	7.5	[4.8%, 11.5%]
Asir	231	17	7.4	[4.6%, 11.5%]
Other*	187	13	7.0	[4.1%, 11.5%]
Hail	112	5	4.5	[1.9%, 10.0%]
Jazan	221	6	2.7	[1.3%, 5.8%]

Note. Percentages and confidence intervals are calculated among cases with non-missing bystander CPR classification under the strict definition. “Other” combines Al Bahah, Al Jouf, Najran, and North Border due to sparse counts. Regional analyses are based on 4,172 records with non-missing harmonized regional information

### Predictors of bystander CPR (multivariable model)

In the complete-case multivariable logistic regression analysis (*N* = 4,267), witnessed cardiac arrest was strongly associated with higher odds of bystander CPR (adjusted odds ratio [aOR] = 5.15, 95% CI [4.20, 6.31], *p* < .001). Longer EMS response time was associated with lower odds of bystander CPR (aOR = 0.98 per minute, 95% CI [0.97, 1.00], *p* = .020). Male sex was associated with increased odds of bystander CPR compared with female sex (aOR = 1.26, 95% CI [1.03, 1.53], *p* = .025). Age and nationality were not independently associated with bystander CPR.

**Fig. 2:**
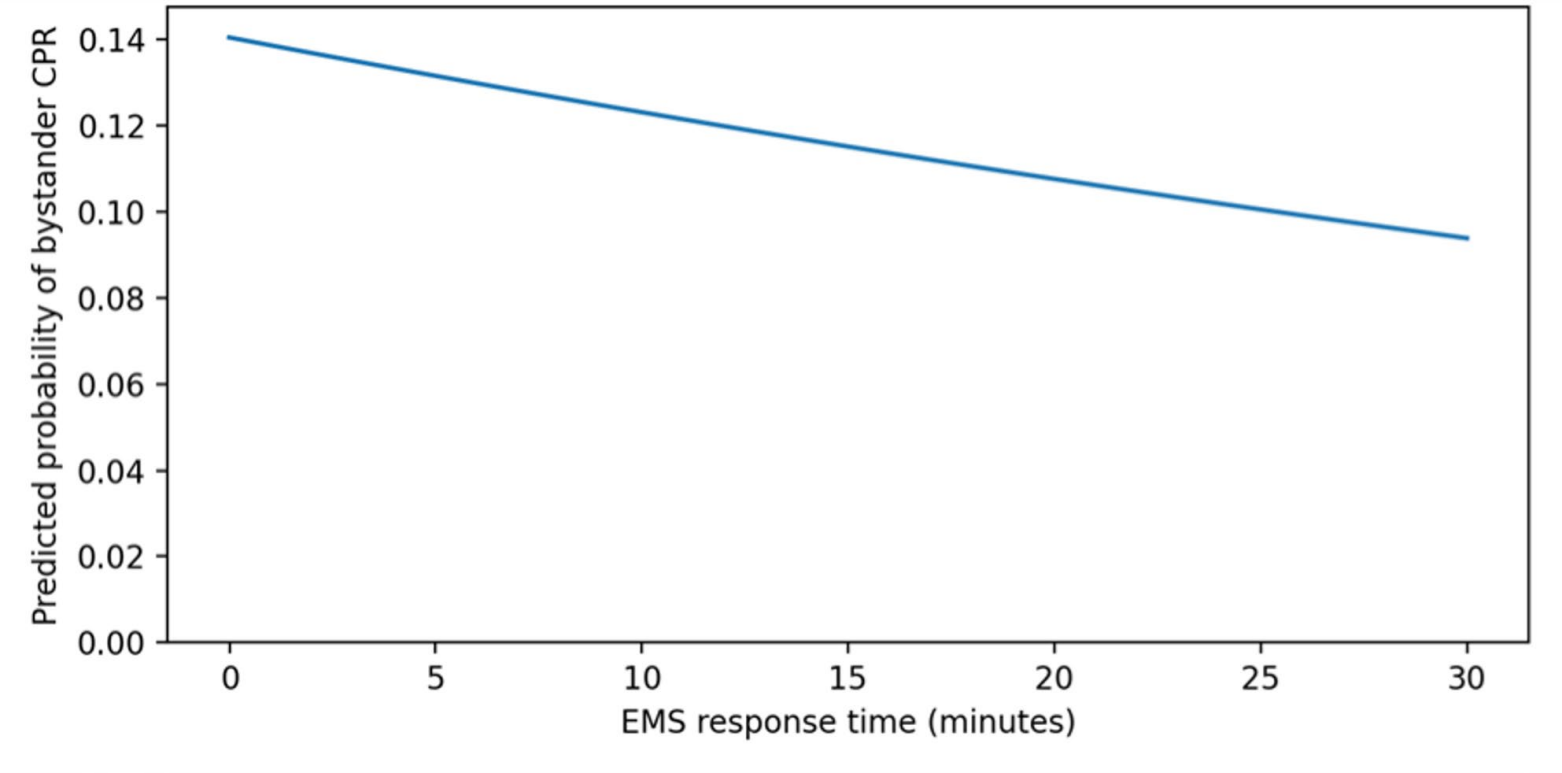
Predicted probability of bystander CPR vs. EMS response

After adjustment, multiple regions demonstrated significantly lower odds of bystander CPR compared with Riyadh (Table 4). Model discrimination was acceptable (AUC = 0.777). The modeled inverse association between EMS response time and the predicted probability of bystander CPR is illustrated in Fig. 2.

**Table 4:** Multivariable logistic regression analysis of predictors of bystander CPR

Predictor	aOR	95% CI	*p*
Age (per year)	1.00	[1.00, 1.01]	0.219
Response time (per minute)	0.98	[0.97, 1.00]	0.020
Sex: Male (vs. Female)	1.26	[1.03, 1.53]	0.025
Nationality: Saudi (vs. Non-Saudi)	0.87	[0.59, 1.26]	0.458
Witnessed (Yes vs. No)	5.15	[4.20, 6.31]	< 0.001
Region: ALQASSIM (vs. RIYADH)	0.33	[0.19, 0.57]	< 0.001
Region: ASIR (vs. RIYADH)	0.27	[0.16, 0.46]	< 0.001
Region: EASTERN REGION (vs. RIYADH)	0.30	[0.22, 0.41]	< 0.001
Region: HAIL (vs. RIYADH)	0.25	[0.10, 0.64]	0.004
Region: JAZAN (vs. RIYADH)	0.14	[0.06, 0.33]	< 0.001
Region: MADINAH (vs. RIYADH)	0.46	[0.32, 0.65]	< 0.001
Region: MAKKAH (vs. RIYADH)	0.34	[0.27, 0.42]	< 0.001
Region: Other (vs. RIYADH)	0.26	[0.14, 0.47]	< 0.001
Region: TABUK (vs. RIYADH)	0.48	[0.22, 1.04]	0.062

Note. aOR = adjusted odds ratio; CI = confidence interval. “Other” combines Al Bahah, Al Jouf, Najran, and North Border due to sparse counts (< 50 each). Reference categories: Female, Non-Saudi, Unwitnessed, and Riyadh

### Sensitivity analysis

To assess the robustness of regional associations, a sensitivity analysis was conducted excluding regions with the highest proportions of unclassifiable outcomes (Table 5). The direction and magnitude of associations remained consistent with the primary model. Witnessed arrest remained the strongest predictor of bystander CPR (aOR ≈ 5), and regional disparities persisted after adjustment. These findings indicate that the observed geographic variation in bystander CPR is unlikely to be explained solely by differences in documentation completeness.

**Table 5:** Sensitivity analysis of regional predictors of bystander CPR after excluding regions with high outcome missingness

Predictor	Adjusted OR	95% CI	*p*-value
Age (per year)	1.00	1.00–1.01	0.219
Response time (per minute)	0.98	0.97–1.00	0.020
Male sex (vs. female)	1.25	1.02–1.53	0.028
Saudi nationality (vs. non-Saudi)	0.88	0.60–1.27	0.472
Witnessed arrest	5.10	4.15–6.28	< 0.001
Region: Al Qassim	0.34	0.20–0.58	< 0.001
Region: Asir	0.28	0.17–0.47	< 0.001
Region: Eastern Region	0.31	0.23–0.42	< 0.001
Region: Hail	0.26	0.11–0.65	0.004
Region: Jazan	0.15	0.07–0.34	< 0.001
Region: Madinah	0.47	0.33–0.66	< 0.001
Region: Makkah	0.35	0.28–0.43	< 0.001
Region: Tabuk	0.49	0.23–1.05	0.067

Note. This sensitivity model excludes regions with the highest proportions of unclassifiable bystander CPR outcomes. Results remained consistent with the primary model, indicating that regional disparities in bystander CPR were not solely attributable to differences in documentation completeness

## Discussion

This multiregional registry-based analysis of SRCA-attended OHCA cases provides a comprehensive overview of bystander cardiopulmonary resuscitation engagement in Saudi Arabia and identifies key patient-, event-, and system-level factors associated with early resuscitation efforts. As the registry reflects OHCA cases documented within the SRCA EMS system rather than all OHCA events occurring nationally, the findings should be interpreted within the context of EMS-attended and transport-associated cases. Using a strict documentation-based definition, bystander CPR was observed in 12.3% of classifiable cases during the first half of 2024. Although this proportion is lower than that reported in many mature OHCA systems, where bystander CPR rates often exceed 40–60% in high-performing settings, it should be interpreted in light of the conservative outcome definition applied and underscores a substantial opportunity to strengthen early community response.

An additional methodological consideration relates to the completeness of documentation used to classify bystander CPR. Under the strict documentation-based definition applied in this study, 17.4% of cases were categorized as unclassifiable due to the absence of explicit attribution of CPR initiation prior to SRCA arrival. Diagnostic comparisons indicated that these cases differed modestly from classifiable cases across several characteristics, including region, EMS response time, and witnessed status. This finding suggests that variations in documentation practices may partly influence estimates of bystander CPR prevalence and geographic variation. Accordingly, the observed regional differences should not be interpreted solely as reflecting true variation in bystander behavior, as differences in EMS configuration, dispatch practices, urban–rural structure, and regional case capture may also have contributed to the observed patterns. The proportion of cases classified as missing or unclassifiable under the strict documentation-based definition (17.4%) further underscores the importance of documentation integrity in registry-based analyses. Strengthening registry completeness and harmonization may therefore represent an additional, often underappreciated, lever for improving OHCA awareness and system performance, adopting internationally validated templates such as the Utstein OHCA reports [16].

The strong association between witnessed arrest and bystander CPR observed in this study is consistent with the established role of witness presence as a prerequisite for early intervention [7, 17–18]. Witnessed status likely reflects both physical proximity to the victim and the ability to recognize cardiac arrest promptly, enabling timely initiation of CPR. The magnitude of this association highlights the importance of early recognition and activation processes and reinforces the potential value of interventions aimed at supporting witnesses at the moment of arrest, particularly through structured DA CPR protocols.

However, the SRCA registry did not contain sufficiently detailed operational data regarding the frequency, consistency, or regional implementation of dispatcher-assisted CPR delivery, nor information regarding dispatch center configuration or triage standardization [19]. Consequently, the present study could not evaluate the direct contribution of DA-CPR practices to regional variation in bystander CPR.

Male sex was also associated with higher odds of bystander CPR, although the magnitude of this effect was modest. This finding should be interpreted with caution, as sex is unlikely to represent a direct causal determinant. Rather, it may reflect underlying contextual and sociocultural factors, such as differences in arrest location, patterns of social interaction, or willingness to initiate physical contact in emergency situations. In some contexts, cultural norms or concerns related to physical contact may influence the likelihood of providing CPR, particularly for female patients. However, as these factors were not directly measured in the present study, this association should be considered exploratory and warrants further investigation.

An inverse association between EMS response time and the likelihood of bystander CPR was also identified. This finding does not suggest a causal effect of response time itself, but rather likely reflects underlying contextual factors. Arrests occurring in less populated, less supervised, or more remote environments may simultaneously be associated with longer EMS response times and a reduced probability of immediate bystander intervention. Similar time-dependent patterns have been described in widely used OHCA survival frameworks, in which early recognition, rapid bystander action, and timely professional response interact dynamically to influence outcomes [20]. From a system perspective, this finding emphasizes the importance of strengthening early links in the chain of survival, particularly in settings where professional response may be delayed.

Regional differences in documented bystander CPR persisted after multivariable adjustment, suggesting that geographic variation may not be explained solely by patient characteristics or event-level factors. Similar regional differences in early resuscitation response have been reported in other OHCA systems [21–22] and are increasingly recognized as manifestations of broader system-level variation rather than individual willingness alone [23]. However, the observed regional patterns should be interpreted cautiously, as differences in documentation practices, EMS organization, dispatch systems, urban–rural structure, and regional case capture may also have contributed to the observed variation. Differences in population density, CPR training penetration, public awareness, dispatcher recognition practices, and EMS organization may all contribute to the observed patterns. In several regions, the absolute number of documented bystander CPR cases was relatively small, which may have reduced estimate precision and increased uncertainty around some regional comparisons, particularly in regions with wide confidence intervals. However, the overall pattern of regional variation remained consistent in the sensitivity analyses, supporting the robustness of the broader geographic trends observed.

Beyond individual-level factors, the observed regional gradient may also reflect structural differences between predominantly urban and more rural or geographically dispersed regions. Urban centers such as Riyadh are characterized by higher population density, shorter average EMS travel distances, and potentially greater exposure to public health campaigns and CPR training initiatives. In contrast, rural or less densely populated regions may face longer response intervals, fewer training opportunities, and lower saturation of community-level preparedness programs. In addition, differences in dispatch organization and the degree of centralization of emergency call centers may influence the consistency and timeliness of DA-CPR delivery. Regions demonstrating higher documented bystander CPR rates may benefit from structured DA-CPR protocols, standardized call scripts, centralized dispatch oversight, and integrated quality monitoring systems, which could serve as scalable models for lower-performing regions. Variation in the density and institutionalization of CPR training programs across regions could further contribute to the uneven distribution of early resuscitation engagement. These hypotheses warrant prospective evaluation but highlight that regional disparities likely reflect broader system configuration rather than differences in individual willingness alone.

The findings of this study have important implications for public health and EMS policy. Evidence-based interventions shown to increase bystander CPR engagement include the systematic implementation of dispatcher-assisted CPR, which can empower untrained or uncertain witnesses to initiate life-saving actions [11, 24]. Nationwide CPR training initiatives, particularly those embedded in schools, workplaces, and community settings, have also been associated with substantial improvements in bystander response and survival at the population level [25]. In addition, EMS system-of-care frameworks that emphasize continuous performance monitoring, feedback, and coordination across dispatch, prehospital, and community components have been shown to reduce variability and improve outcomes [19, 26]. Emerging strategies, such as mobile lay-responder activation systems, may further enhance early CPR initiation, especially in public locations and densely populated areas [27–28].

In Saudi Arabia, ongoing national efforts to strengthen early resuscitation response have included expansion of public CPR training initiatives, increasing integration of CPR education within community and institutional settings, and broader discussion regarding dispatcher-assisted CPR and community responder activation strategies. However, the implementation, penetration, and operational maturity of these initiatives may still vary substantially across regions, potentially contributing to the geographic differences observed in the present study.

## Limitations

Although based on a large multiregional cohort of OHCA cases attended by the SRCA in Saudi Arabia, these real-world EMS registry data allow only limited conclusions. The SRCA registry reflects OHCA cases documented within the SRCA EMS system and does not necessarily capture all OHCA events occurring nationally. In addition, cases in which resuscitation was not initiated, including some patients declared dead on EMS arrival, were excluded from the analytical cohort. Consequently, the findings should be interpreted as representative of EMS-attended and transport-associated SRCA cases rather than the full national OHCA burden.

The registry did not contain sufficiently detailed operational information regarding the specific criteria used to withhold resuscitation or declare death on EMS arrival, limiting further assessment of potential selection effects related to exclusion of non-resuscitated cases. Similarly, the registry did not provide a fully standardized operational definition of EMS response time across all regions, which may have introduced minor measurement variability in analyses involving response-time estimates.

Because hospital-level outcomes were unavailable, no correlations could be assessed regarding survival or neurological outcome. Even after adjustment for multiple covariates, residual confounding due to factors such as arrest location (public versus private places), availability and use of automated external defibrillators, quality and continuity of CPR, dispatcher-assisted CPR delivery, dispatcher recognition of cardiac arrest, and urban–rural context could not be evaluated and may have influenced bystander response.

The strict documentation-based definition of bystander CPR improved specificity and reduced potential outcome misclassification; however, this conservative strategy may have underestimated the true prevalence of bystander CPR, particularly when incomplete or ambiguously documented reports were excluded. Variability in documentation practices across regions may also have influenced estimates of regional variation and introduced selection bias. In addition, because the multivariable regression analysis used a complete-case approach, exclusion of records with missing predictor information may have introduced additional selection bias if missingness was not completely random. In several regions, the absolute number of documented bystander CPR cases was relatively small, which may have reduced estimate precision and increased uncertainty around some regional comparisons.

Finally, the study was limited to a six-month observation period (January–June 2024), and potential seasonal variation in OHCA incidence, EMS demand, or bystander behavior could not be evaluated. Consequently, seasonal effects may have influenced estimates of bystander CPR prevalence and regional comparisons.

Despite these limitations, the study provides valuable multicenter EMS registry evidence on early resuscitation processes and regional variation within the SRCA EMS context in Saudi Arabia. The findings offer a foundation for targeted quality improvement initiatives and future research incorporating more detailed prehospital and in-hospital outcome data.

## Conclusions

Bystander cardiopulmonary resuscitation remains infrequently performed in OHCA cases attended by the SRCA in Saudi Arabia, with pronounced and persistent regional disparities. Using a conservative, documentation-based definition, this study suggests that early bystander intervention is strongly associated with witnessed arrest and is less likely in settings characterized by longer EMS response times, underscoring the critical importance of the earliest links in the chain of survival.

The substantial geographic variation observed after multivariable adjustment indicates that differences in bystander CPR engagement may be driven primarily by system-level factors rather than patient characteristics alone. These findings highlight the need to prioritize standardized DA-CPR, expand population-level CPR training, and implement region-specific performance monitoring and quality improvement strategies within EMS systems in Saudi Arabia. Strengthening early recognition, documentation, and community engagement represents an important opportunity to reduce disparities and improve early resuscitation response across SRCA-attended settings.

## Data Availability

The data analyzed in this study were obtained from the Saudi Red Crescent Authority OHCA registry and are not publicly available due to institutional and privacy restrictions. De-identified data may be available from the corresponding author upon reasonable request and with permission from the Saudi Red Crescent Authority.

## Abbreviations

OHCA: Out-of-Hospital Cardiac Arrest
EMS: Emergency Medical Services
CPR: Cardiopulmonary Resuscitation
DA-CPR: Dispatcher-Assisted Cardiopulmonary Resuscitation
SRCA: Saudi Red Crescent Authority
SD: Standard Deviation
CI: Confidence Interval
aOR: Adjusted Odds Ratio
AUC: Area Under the Receiver Operating Characteristic Curve
